# The road to cancer control goes through leukemia research

**DOI:** 10.3747/co.v16i5.480

**Published:** 2009-09

**Authors:** E.J. Freireich

Lay people, particularly people in the press, frequently ask the question “When will we cure cancer?” I believe that we will **never** cure cancer.

For an organism like a human being, which begins as one cell and, in the adult phase, averages 70 trillion (70,000,000,000,000) cells, with these cells continuously dying and being replaced, it is apparent that this mind-boggling rate of reproduction will almost certainly have errors. We know that cancer is strongly associated with age—that is, the longer an organism lives, the higher the probability that a malignancy will develop—and the theoretic basis for this fact is that it takes multiple events to initiate a malignancy. We also know that patients with a diagnosed cancer that is eliminated or cured are at substantially increased risk for developing another malignancy. Again, theoretically, because one “hit” has already occurred, a second “hit” that might not initiate a cancer in an otherwise normal person could initiate a cancer in a patient who has had a previous cancer.

Thus, as we prolong life, the greater is the likelihood of a malignancy developing. And so one has to reach the conclusion that cancer, as an umbrella term for a large group of malignancies, will always be present and will never be eradicated in the same sense that polio or smallpox can be eradicated. Rather, the malignancies that occur can be cured, and consequently life will be prolonged and cancer will be considered controlled, particularly if other events such as other diseases or accidents or homicide or suicide terminate people’s lives.

A very important concept that is highly popular with lay people, and particularly with politicians, is that the way to control cancer or even to eliminate it is to use the current methods of early detection. The hypothesis that early detection is a technique for controlling cancer is based entirely on the Halstedian hypothesis that cancer begins in a site, spreads regionally, and then spreads systemically. Unfortunately, when tested, this hypothesis has always failed, as shown most conclusively in studies of therapy for breast cancer^1^. The application of more and more radical surgery does not in fact increase the cure rate of diseases such as breast cancer, prostate cancer, and so on, as is dramatically demonstrated by the data that have been carefully summarized in an important book titled *Should I Be Tested for Cancer*? That book demonstrates that the early detection model simply inclines to lead-time bias and recognition of abnormalities that would never become systemic cancer^2^. The latter statement is even more strongly supported by the fact that, for diseases for which excellent early detection techniques are available, such as breast cancer and prostate cancer, the incidence of cancer in developed countries is very much higher than it is in countries such as Africa, eastern Europe, and Micro-Polynesia. Yet the mortality in those areas is comparable to the mortality in North America^3^. One therefore has to conclude that the early detection model will not control cancer, but simply increase the incidence of cancer in addition to introducing very powerful lead-time bias.

Everyone is willing to concede that prevention could be an effective strategy for eliminating cancer. Certainly, eliminating the three most important proven carcinogens—tobacco, ultraviolet exposure, and alcohol—is certainly effective in reducing the frequency of cancer. Yet, more than 50 years ago, it was conclusively shown that smoking is responsible for a great preponderance of human lung cancer, and although we have been slightly successful in reducing smoking practices in the male population, the reduction approach has been frustratingly ineffective in the female population, in which smoking continues to increase. But it has been observed in male populations that the proportion of patients who are smokers among those diagnosed with lung cancer continues to decrease. Clearly, then, tobacco as a carcinogen is a promoting agent and not an initiating agent for lung cancer.

The illusion that further research on prevention or early detection will eliminate cancer is just not compatible with the science and the facts that we have at hand. What is obvious is that, to control cancer, we will have to learn to control advanced cancer—that is, systemic metastatic cancer. That disease is the cause of morbidity and mortality, and if we are going to control systemic cancer, I contend that we should focus our attention on leukemia. Given that leukemia is a systemic disease in every patient, we do not have to deal with the local–regional–systemic hypothesis that involves surgery, radiation, and modalities that are designed to control local disease and to prevent metastatic disease. Leukemia is not helped by surgery or radiation, and local control plays no role. So we have to study the systemic form of leukemia from the outset. Even more important is the fact that leukemia is much more accessible to the scientific community than any other form of malignancy. The cancer cells are in the blood and in the bone marrow, and both of these organs can be sampled repeatedly with enormous quantities of material, so that questions of biology can be addressed in the laboratory. Even more important is the fact that human leukemia cell lines have been established and many more are being established every day: one can study the entire malignant process *in vitro*, and transplants of human leukemia into experimental animals allow for the study of leukemic cells *in vivo* in those animals.

Finally, and perhaps most important, dramatic progress has been made in the control of leukemia over the last 60 years: the discovery of combination chemotherapy, the discovery of adjuvant chemotherapy, the discovery of neoadjuvant chemotherapy (that is, systemic therapy before local therapy for localized tumours), the discovery of the genetic basis of leukemia (the chromosome abnormalities, the molecular abnormalities). Most important is the fact that, for childhood acute lymphoblastic leukemia, 85% of patients achieve long-term survival—more than 5 years. Adults with acute lymphoblastic leukemia are increasingly benefiting from therapy, and 20% of patients with acute myeloblastic leukemia experience survival in excess of 5 years. Patients with chronic granulocytic leukemia have seen their disease totally transformed—from one that was 100% fatal, with median survival of 3.5 years, to a circumstance in which more than 90% are free of disease at 10 years as the result of a therapy in which a pill is taken orally. In chronic lymphocytic leukemia, there has been a dramatic improvement in treatment that not only controls the disease, but extends survival. For the other leukemic disorders—the myeloproliferative diseases and the myelodysplastic diseases-again, enormous advances in understanding and treatment have occurred in the last five years. Much of this advancement has been extensively reviewed in the literature and need not be reviewed here.

In sum, I believe that control of cancer as a public health problem will result not from early detection, but from control of systemic, metastatic disease. Leukemia is an excellent model for understanding human metastatic disease, and the progress that has been made in the control of leukemia has been immediately applicable to the other, more common solid tumours in humans. We should therefore increasingly focus our scientific efforts on controlling human leukemia as the best strategy for controlling cancer as a major public health problem.
